# Intraoperative Applications of Artificial Intelligence in Robotic Surgery: A Scoping Review of Current Development Stages and Levels of Autonomy

**DOI:** 10.1097/SLA.0000000000005700

**Published:** 2022-09-30

**Authors:** Baptiste Vasey, Karoline A.N. Lippert, Danyal Z. Khan, Mudathir Ibrahim, Chan Hee Koh, Hugo Layard Horsfall, Keng Siang Lee, Simon Williams, Hani J. Marcus, Peter McCulloch

**Affiliations:** * Nuffield Department of Surgical Sciences, University of Oxford, Oxford, UK; † Department of Engineering Science, Institute of Biomedical Engineering, University of Oxford, Oxford, UK; ‡ Critical Care Research Group, Nuffield Department of Clinical Neurosciences, University of Oxford, Oxford, UK; § Institute of Psychiatry, Psychology and Neuroscience, King’s College London, London, UK; ∥ Department of Neurosurgery, National Hospital for Neurology and Neurosurgery, London, UK; ¶ Wellcome/EPSRC Centre for Interventional and Surgical Sciences, University College London, London, UK; ** Bristol Medical School, Faculty of Health Sciences, University of Bristol, Bristol, UK; # Department of General Surgery, Maimonides Medical Center, Brooklyn, NY

**Keywords:** artificial intelligence, autonomy, evaluation, IDEAL, intraoperative, machine learning, outcomes, performance, robotic, surgery

## Abstract

**Objective:**

A scoping review of the literature was conducted to identify intraoperative artificial intelligence (AI) applications for robotic surgery under development and categorize them by (1) purpose of the applications, (2) level of autonomy, (3) stage of development, and (4) type of measured outcome.

**Background:**

In robotic surgery, AI-based applications have the potential to disrupt a field so far based on a master-slave paradigm. However, there is no available overview about this technology’s current stage of development and level of autonomy.

**Methods:**

MEDLINE and EMBASE were searched between January 1, 2010 and May 21, 2022. Abstract screening, full-text review, and data extraction were performed independently by 2 reviewers. The level of autonomy was defined according to the Yang and colleagues’ classification and stage of development according to the Idea, Development, Evaluation, Assessment, and Long-term follow-up framework.

**Results:**

One hundred twenty-nine studies were included in the review. Ninety-seven studies (75%) described applications providing Robot Assistance (autonomy level 1), 30 studies (23%) application enabling Task Autonomy (autonomy level 2), and 2 studies (2%) application achieving Conditional autonomy (autonomy level 3). All studies were at Idea, Development, Evaluation, Assessment, and Long-term follow-up stage 0 and no clinical investigations on humans were found. One hundred sixteen (90%) conducted in silico or ex vivo experiments on inorganic material, 9 (7%) ex vivo experiments on organic material, and 4 (3%) performed in vivo experiments in porcine models.

**Conclusions:**

Clinical evaluation of intraoperative AI applications for robotic surgery is still in its infancy and most applications have a low level of autonomy. With increasing levels of autonomy, the evaluation focus seems to shift from AI-specific metrics to process outcomes, although common standards are needed to allow comparison between systems.

On April 11, 1985, Kwoh et al^1^ performed the first robot-assisted surgical procedure, a stereotactic brain biopsy with the UNIMATION PUMA 200. Despite this initial attempt at developing supervisory-controlled robot, and with few exceptions ever since, most robot-assisted surgery has then evolved based on a master-slave paradigm, whereby the surgical robot stays at all times completely under the control of the operator.

The progress in available computational power and machine learning (ML) mathematical models over the past years has challenged this status quo. Applications based on artificial intelligence (AI) have become increasingly popular in robotic surgery,^2,3^ with long-term objectives ranging from autonomously performing basic routine tasks, like suturing, to independently conducting advanced surgeries. In the shorter term, AI intraoperative applications for robotic surgery identified by previous reviews as under development include target and anatomic structure identification, instrument tracking and navigation (including skills transfer), instrument control and improved feedback, low-level automated tasks (eg, knot tying), surgical step segmentation and alerting, performance monitoring and training, and optimization of the human–robot interaction.^3–6^

AI-based intraoperative applications for robotic surgery offer opportunities to improve the efficacy, safety, and efficiency of procedures, but they also introduce new obstacles related to validation, approval, and trust of the AI applications and supporting robotic systems. One of the key considerations under this new paradigm is the AI application’s targeted level of autonomy; AI being a tool to achieve a desired level of autonomy for a task with a given complexity. The performance of an AI application and its safety profile can indeed only be fully appraised in the context of the desired level of autonomy for a specific task. For example, an AI system identifying the correct excision target 90% of the time might be considered good enough for an application with conditional autonomy (humans have to validate every action plan), but not for an high autonomy application (humans only have an occasional supervisory role). Several scales have been proposed to classify the level of autonomy of surgical robots,^2,7,8^ mostly ranging from no support at all (humans are in control of every information processing, decision-making, and action execution) to full robot autonomy (humans play no role in the procedure).

To date, surgical robots are considered as medical devices and mainly regulated through the Food and Drug Administration and European CE marking directives. However, there is no clear scientific framework on how they should be evaluated, let alone their AI components. In this context, the IDEAL collaboration (Idea, Development, Evaluation, Assessment, and Long-term follow-up), an initiative dedicated to improving the evaluation of complex interventions, has convened the IDEAL Robotic Colloquium. The Colloquium is an international and multistakeholder consensus process whose main objective is to develop an adaptation of the IDEAL framework for the evaluation of surgical robots. The initial IDEAL framework is a 5-stage development and evaluation pathway for surgical innovation, considering surgical procedures as complex interventions.^9,10^ Stage 1 describes the first in human evaluation, stages 2a and 2b the subsequent refinement of the procedure and exploration of its clinical utility under different operators and implementation settings, stage 3 the multicentric comparative studies, and stage 4 the long-term follow-up and surveillance. More recent modifications and extensions, like IDEAL-D and IDEAL stage 0, are also relevant to this work, because they provide specific guidance for surgical devices and introduce recommendations for their preclinical evaluation.^11,12^

To inform the Colloquium’s discussion on AI and autonomous functions in robotic surgery, it was important to explore the scope of AI applications under development and the methodologies used to evaluate them. This study aimed to identify the different intraoperative AI applications in robotic surgery and categorize them by (1) purpose of the applications, (2) level of autonomy (as defined by Yang et al^8^), and (3) stage of development, as defined by the IDEAL framework. Secondary objectives were to identify the mathematical models most commonly used to train these applications and the most commonly used methodologies to evaluate them. By doing so, we hope to provide an insight into the current status of intraoperative AI-based applications in robotic surgery, their future prospects in surgery, and how distant these are from routine clinical use. In addition, this would highlight potential risks and shortcomings in the innovation pathway of this relatively new technology.

## METHODS

This scoping review was conducted and reported in accordance with the Arksey and O’Malley’s framework for scoping reviews, the subsequent amendments made by the Joanna Briggs Institute, and Preferred Reporting Items for Systematic Reviews and Meta-Analyses extension for Scoping Reviews^13–15^ The Protocol was registered on the Open-Science Framework on March 9, 2021, with DOI 10.17605/OSF.IO/WXQ9Y.

### Literature Search

MEDLINE and EMBASE were searched on November 26, 2020, using a piloted search strategy (Supplementary Note 1, Supplemental Digital Content 1, http://links.lww.com/SLA/E229). To include the most up-to-date data, a search update was conducted on May 21, 2022. The search windows reached back to January 1, 2010 (peer-reviewed articles) and January 1, 2018 (conference abstracts). Inclusion criteria, all of which needed to be fulfilled, were original research in English, describing an intraoperative AI-based application for robotic surgery, or the preclinical evaluation of an AI application whose main purpose is to be used intraoperatively. Exclusion criteria were reviews or comments, studies describing radiosurgery applications, studies describing application for robot-assisted straight needle injection only, and studies whose full text was not available.

For the purpose of this review, applications were considered as AI-based if their main decision-making components relied on ML algorithms. ML was defined as mathematical models having the ability to independently learn, from input data, knowledge unknown to their programmers and to generate outputs that had not been explicitly programmed.^16,17^ We defined surgical procedures as procedures performed for the purpose of structurally altering the human body by the incision or destruction of tissues (American Medical Association, Definition of Surgery H-475.983) after gaining epithelial or endothelial access. Intraoperative was defined as the period from the first epithelial or endothelial access opening to the closure of the last access point. Additional sources and gray literature were also searched, including the reference list of similar reviews in the field, a forward literature search of studies included after the initial literature search (ie, review of all citing articles referenced on the Web of Science platform), the clinical trial registrations on the Cochrane Central Register of Controlled Trials (CENTRAL), ClinicalTrials.gov, and the EU Clinical Trials Register. The modified search strategy for trial registers can be found in Supplementary Note 2, Supplemental Digital Content 1, http://links.lww.com/SLA/E229.

### Abstracts and Full Texts Screening

For the initial literature search, all search records were imported into EndNote X8 (Thomson Reuters) for deduplication and exclusion of publication in another language than English. All abstracts were independently screened by at least 2 reviewers (K.A.N.L., B.V., M.I., C.H.K., H.L.H.), using Rayyan.^18^ Disagreements were adjudicated by a third reviewer (B.V., M.I.). Full-text screening was independently conducted by at least 2 reviewers (K.A.N.L., B.V., M.I., C.H.K., H.L.H.) and conflicts adjudicated by a third reviewer (B.V., M.I.). For the search update, abstracts and full texts were screened by a single reviewer (S.W.) and unclear cases adjudicated by a second reviewer (B.V.).

### Data Extraction

The following data were extracted, using a piloted extraction form: (1) year of publication, (2) surgical specialty (defined as per Royal College of Surgeon England) and type of procedure, (3) name of the robotic system used and primary task of the AI-based application, (4) level of autonomy of the AI application, using the 6 levels scale defined by Yang et al,^8^ (5) type of ML model used and training set data type, (6) study design, (7) primary outcome evaluated, and (8) stage of evaluation as defined by the IDEAL framework and IDEAL stage 0 extension.^9,10,12^ The extraction was independently conducted by at least 2 reviewers (K.A.N.L., B.V., M.I., C.H.K., H.L.H., K.S.L.) and conflicts were adjudicated by a third reviewer (K.A.N.L., B.V.). For the search update, extraction was conducted by a single reviewer (S.W.) and reviewed by a second reviewer (B.V.).

### Data Analysis

Frequency count and narrative summaries were produced for each of the study’s main results. AI applications were categorized according to an existing framework by Kassahun et al^5^: event detection (visual recognition of critical structures or incidents), environment modeling (registration and reconstruction of surroundings), localization (visual detecting an object within space), planning (automated trajectory design and control), robot control (coordination of robot movements), and skills analysis (assessment of performance). Measured outcome metrics were organized into procedural and AI-based, with the latter subdivided into the specific AI task being evaluated (classification, clustering, object detection, and regression). Given the heterogeneity in almost all facets of the included studies, no quality assessment was performed. Graphics were produced using Excel (version 16.65, Microsoft) and Visme (Visme, accessed January 2, 2021).

## RESULTS

### General Search Characteristics

**FIGURE 1:**
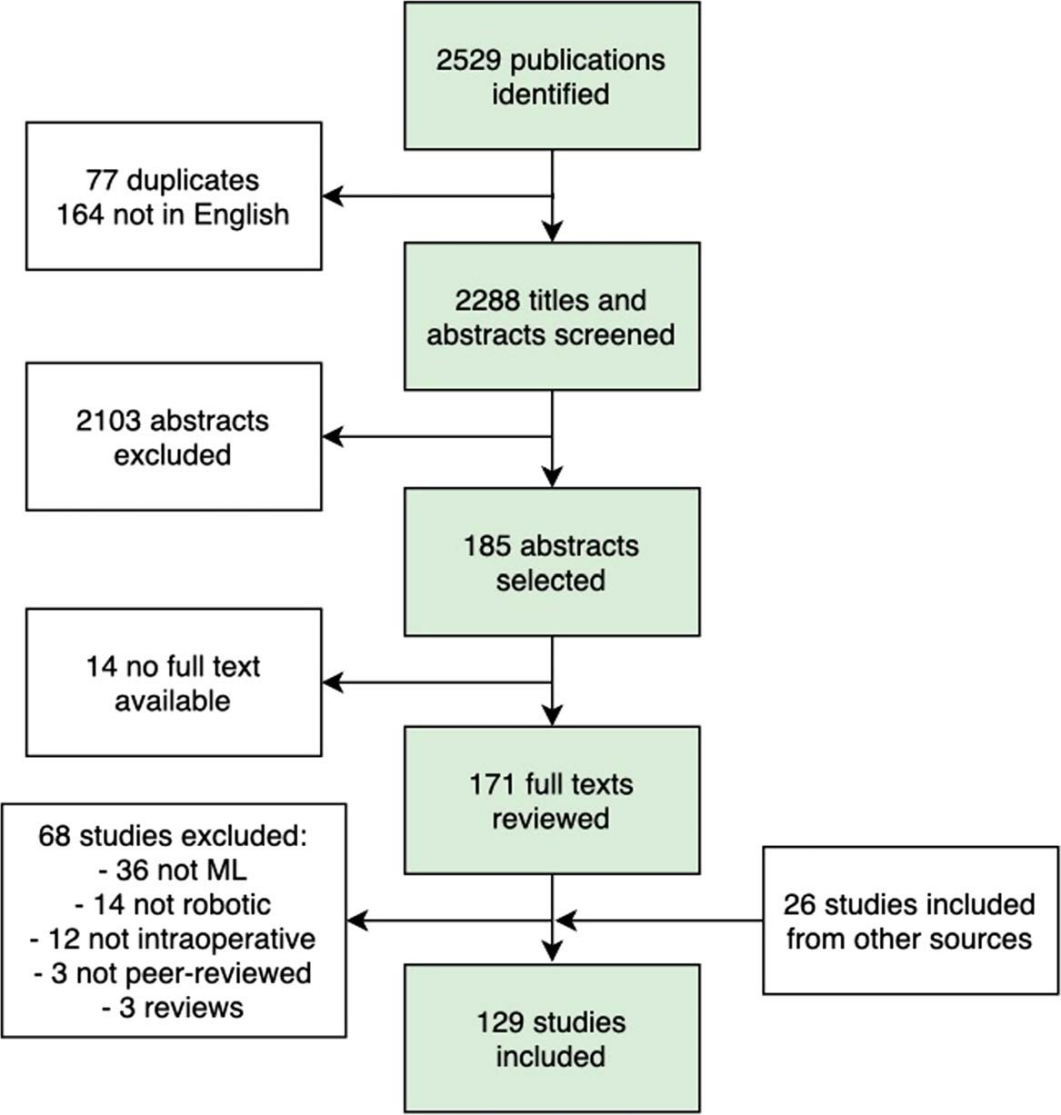
PRISMA flowchart. Other sources include reference lists from similar reviews and expert recommendations.

**FIGURE 2:**
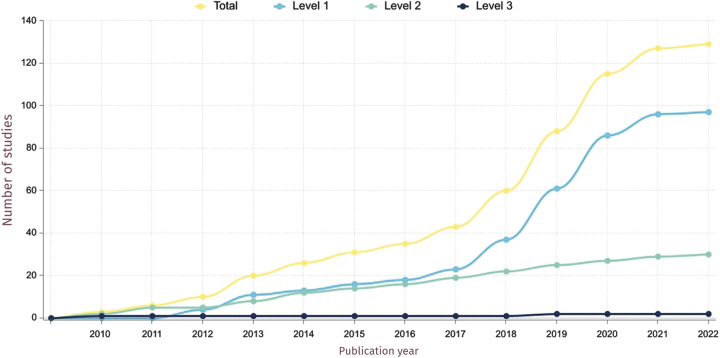
Studies published over time, stratified by level of autonomy of the AI-augmented robotic system studied.

Our search retrieved 2529 peer-reviewed articles and conference abstracts (Fig. 1). After removing duplicates and non–English-language articles, 2288 titles and abstracts were screened. One hundred eighty-five abstracts were selected for full-text review, of which 171 had a full text available. One hundred three studies met all the inclusion criteria, and 26 studies were added from other sources for a total of 129 included studies. There is evidence that this is a rapidly expanding field, with 54% of the included studies published in the last 3 years and a half (Fig. 2).

### General Study Characteristics

In terms of study design, all studies were preclinical in nature (thus all IDEAL stage 0). The majority of studies involved experiments in silico or ex vivo on inorganic material (116/129; 90%). A minority of studies were performed using organic materials ex vivo (9/129; 7%; most commonly porcine) or in vivo (4/129; 3%, in porcine models) (Supplementary Table 1, Supplemental Digital Content 1, http://links.lww.com/SLA/E229). In terms of surgical specialty, most (73/129; 57%) integrated AI robotic applications were assessed in a cross-specialty manner, that is, using universal or cross-disciplinary surgical tasks (eg, suturing) or generic analysis (eg, instrument tracking). Regarding those studies presenting an application for a surgical task specific to a specialty, or claimed a link to a specific specialty, the most frequently observed specialty was general surgery (12/129; 9%), followed by, urology (11/129; 9%), vascular (7/129; 5%), cardiothoracic (7/129; 5%), ophthalmology (5/129; 4%), and neurosurgery (5/129; 4%) (Supplementary Table 1, Supplemental Digital Content 1, http://links.lww.com/SLA/E229). However, some of these specialty-specific studies evaluated applications, which in practice could also be applied to other disciplines, like for example, organ depth or cancer margin estimation.

### Technology Characteristics

In terms of robotic systems, the most common was the Da Vinci (34/129; 26%), followed by the KUKA arm (6/129; 5%), Steady-Hand Eye Robot (4/129; 3%), and Raven 2 (3/129; 2%). Fifty-three studies (41%) did not specify the exact robotic system used (Supplementary Table 1, Supplemental Digital Content 1, http://links.lww.com/SLA/E229). In addition, AI applications were used for a variety of functions, most commonly environment modeling (39/129; 30%), followed by tracking/localization (21/129; 16%), robot control (21/129; 16%), planning (18/129; 14%), event detection (18/129; 14%), and skill analysis (12/129; 9%) (Table 1, Supplementary Table 1, Supplemental Digital Content 1, http://links.lww.com/SLA/E229). The type of AI algorithm used was similarly heterogenous. The most prevalent type of underlying algorithms used were neural networks (75/129; 58%), with other models ranging from simple logistic regressions to support vector machines and Gaussian mixture models. Data used to train the models were obtained from a variety of sources: kinematic data (47/129; 36%), videos (45/129; 35%), images (28/129; 22%), force sensor data (12/129; 9%), audio recording (1/129; 1%), and other data types (4/129; 3%) (Supplementary Table 1, Supplemental Digital Content 1, http://links.lww.com/SLA/E229).

**TABLE 1:** Summary Table of Application of AI Stratified by Level of Autonomy

	Level of Autonomy		
Application of AI to Robotic System	Level 1 Robot Assistance	Level 2 Task Autonomy	Level 3 Conditional Autonomy
Environment modeling	35	4	0
Event detection	16	2	0
Localization/tracking	18	3	0
Planning/navigation	3	13	2
Robot control	13	8	0
Skill analysis	12	0	0
Total	97	30	2

No studies were found describing applications at levels 4 and 5.

### Level of System Autonomy

**FIGURE 3:**
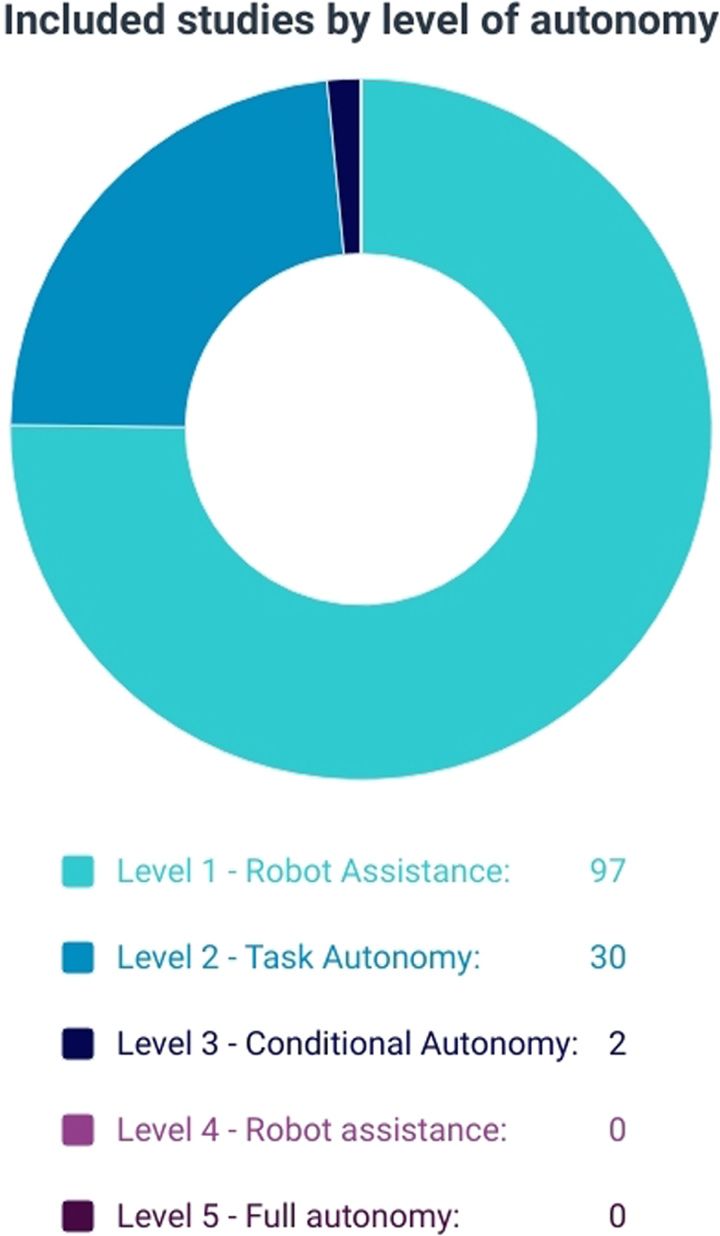
Numbers of studies per the level of autonomy of the AI-augmented robotic system studied.

The majority of studies (97/129; 75%) included AI application for robotic surgery with a low level of autonomy (level 1—Robot Assistance), which has increased exponentially over the last 3 years (Fig. 2). Regarding the 30 studies (23%) that explored systems with Task Autonomy (level 2), the majority of these utilized AI for planning (13/30; 43%) and robot control (8/30; 27%) (Table 1). Only 2 studies (2%) involved applications judged to achieve Conditional autonomy (level 3) (Fig. 3). Tan et al^19^ explored the insertion of a flexible needle via a bespoke robotic supervisory-controlled system, which utilized a universal distributional Q-learning AI algorithm to assist with planning and robot control. This system, assessed via in silico and ex vivo (synthetic liver phantom) demonstrated an ability to reach multiple targets through a single-insertion site. Similarly, De Momi et al^20^ also utilized AI (fuzzy risk model) for path planning and assisted navigation of a flexed probe using the ROBOCAST system during ex vivo neurosurgical procedures.

### Types of Measured Outcomes

**FIGURE 4:**
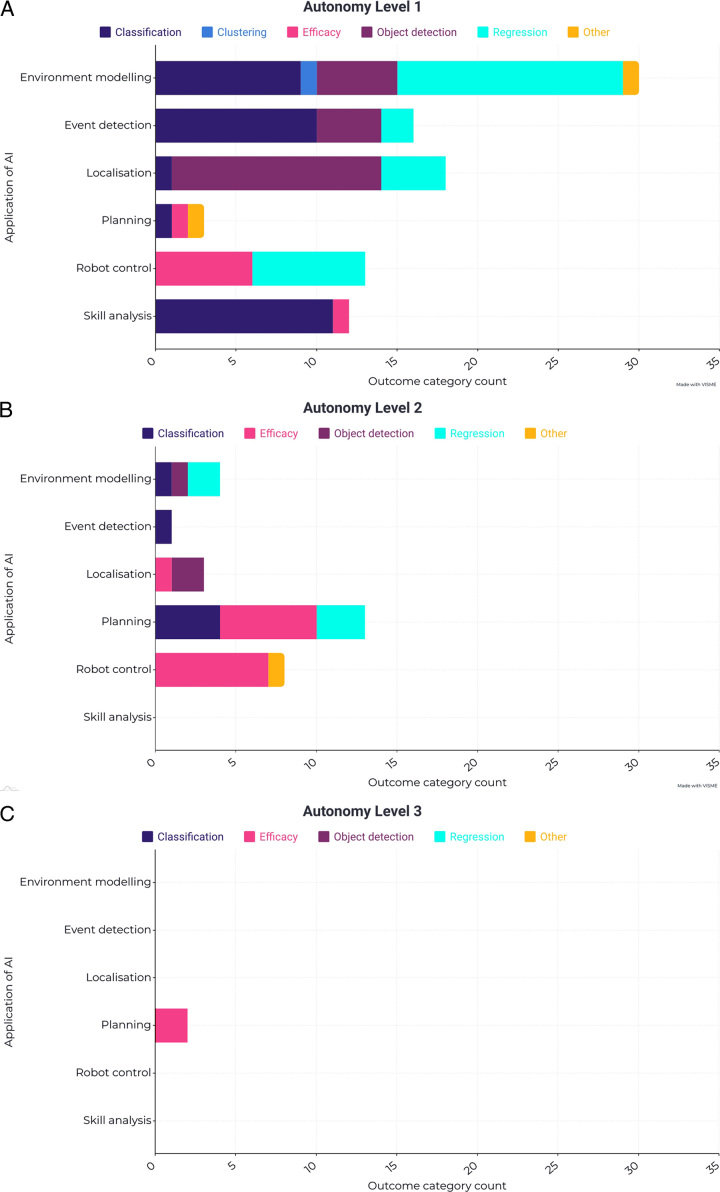
A-C, Stacked bar chart of outcome categories against application of AI, stratified by each level of autonomy.

Measured outcomes focused on performance of the AI component of the AI-augmented robotic system in the majority of studies; most frequently classification (38/129; 29%), object detection (30/129; 23%), and regression (29/129; 22%), with specific metrics within these categories considerably variable (Fig. 4, Supplementary Tables 1, Supplemental Digital Content 1, http://links.lww.com/SLA/E229 and 2, Supplemental Digital Content 1, http://links.lww.com/SLA/E229). Classification outcomes were usually seen in skills analysis, event detection and environmental modeling applications of AI. Regression outcomes were often used in AI applications for environment modeling, localization, robot control, and planning; while object detection was mostly used in localization and event detection applications. Efficacy-based outcomes were used in 28 studies (22%) and reflected either performance of the AI application or directly assessed the procedure performed by the robot—again, encompassing a wide range of metrics, usually specific to the study experiment (Fig. 4, Supplementary Table 2, Supplemental Digital Content 1, http://links.lww.com/SLA/E229). In keeping with the IDEAL stage 0 nature of included studies, all outcome metrics were preclinical.

## DISCUSSION

### Principal Findings

This scoping review of 129 studies provides an overview of the current landscape of intraoperative AI applications for robotic surgery in development, stratified according to level of autonomy and level of development according to the IDEAL framework.

It is evident that intraoperative AI application for robotic surgery is a growing field with particularly rapid expansion over the last 3 to 5 years. Current applications are all in the preclinical (IDEAL stage 0) phases of development.^12^ No clinical studies or evidence of translation of these technologies were identified in this scoping review of literature and clinical trial databases, even after screening publications citing the included studies. This may reflect lack of publishing due to intellectual property reasons, the omission of AI components description when evaluating fully integrated robotic systems, or the relative infancy of this field, which will benefit from the ongoing wider progress of AI and robotics in medicine. Furthermore, the majority of AI applications explored specific functions (eg, instrument tracking, robot control, etc), operating with low levels of autonomy (level 1—“Robot Assistance”). This could reflect the fact that isolated AI applications are unlikely to reach a high level of autonomy. As this field develops, higher levels of autonomy will likely be achieved through interlinking and synergy between these lower autonomy functions.^21^ A recent example of such integration is the Smart Tissue Autonomous Robot, which combined camera control, instrument collision avoidance, tissue motion tracking, landmark detection, suture planning, and several other autonomous functions for laparoscopic intestinal anastomosis on in vivo porcine models.^22^ Such interlinking would also probably shift the evaluation focus from low-level technical performance metrics to procedure level efficacy metrics, as suggested by the higher rate of study using the latter among autonomy level 2 and 3 applications.

Indeed, a successful integration of this technology into clinical workflow will need an evaluation focus going behind traditional performance metrics. The field is now reaching a point where robotic procedures with a higher level of autonomy (at least levels 3 and 4) will soon be technically possible for low-complexity interventions. This could open the way to a wide range of applications such as increased surgical output to reduce waiting lists or local task control to overcome lag issues in telerobotic surgery. However, technical feasibility does not mean acceptance and further changes in mindset and legal framework concerning liability and responsibility will need to take place first.^23^ We and others have argued that human factor considerations and aspects related to safety will also play a pivotal role in gaining practitioners’ and patients’ trust in AI-based systems.^24^ Questions relating to handover procedures, human override, or deskilling of human operators, among others, still must be addressed before surgical robots with higher autonomy level become common components of modern operating rooms.

Such mindset changes will need strong arguments, based on robust, transparent, and comparable evaluation. Our review shows that there is for the time being widespread heterogeneity across almost all facets of the included studies, though. This is partly due to the wide-scoping nature of this review and the rapidly expanding nature of this field—covering various surgical specialties (most commonly cross-specialty) and study designs (most commonly ex vivo or in silico). As a result, the type of AI used (most commonly neural network based), the purpose of AI integration (most commonly environment modeling), and the robotic system used were wide ranging. However, and more importantly, the evaluation metrics used to assess the technology was significantly heterogenous, even across studies on related AI applications. Although diversity of evaluated applications and underlying AI models is a positive reflection of the field’s dynamism, evaluation metrics heterogeneity among studies describing AI applications with similar objectives prevents comparative analysis of performance and safety. Similarly, data reporting was diverse, likely reflecting the lack of a reporting framework to guide this. More generally, there is a clear need for structured guidance on how these applications and supporting robotic systems should be evaluated across their robotic performance, AI integration and interface with humans. In other fields, consensus-driven outcome and measurement sets have been helpful in aligning studies.^25^

Therefore, we are presented with the nascent opportunity to shape and structure the development and evaluation of AI applications for robotic surgery preemptively—as the field exponentially grows and before the demonstrated preclinical technologies begin to enter the clinical setting.

### Finding in Context

There are several reviews of note, which explore related fields. A narrative review by Panesar et al^2^ outlines a framework for generating autonomy in robotic systems using AI, focusing on (1) the system’s ability to sense its environment, (2) interpret this data, and (3) enact appropriate physical tasks, the whole in a dynamic function loop. They highlight the scope of potential impact for these technologies, not only in improving individual outcomes but also in improving access to surgical care, for example, in challenging environments (such as remote terrains and space). Another narrative review by Ma et al,^4^ corroborates AI’s potential to bolster the ability of robotic systems, ranging from anatomy recognition and autonomous tasks to surgical training and assessment. They highlight the importance of the safe curating and storing of the granular multimodal surgical data needed to harness the full potential of AI in these systems.

Moustris et al^6^ explore autonomous robotic surgical systems through a nonsystematic literature review and produce a narrative synthesis of the available robotics systems capable of performing tasks such as suturing, cochlear implantation, and stereotactic radiosurgery with varying levels of autonomy. The review cites the importance of multidisciplinary input for amalgamating low autonomy technologies into high autonomy systems capable of addressing the Autonomy Levels for Unmanned Systems (ALFUS) domains: mission complexity, environmental difficulty, and human independence.^26^ Similarly, Kassahun et al^5^ summarized the ML techniques used in surgical robots and training via literature review. They provide potential categories for the application of AI to facilitate autonomous robotics (which we have adopted in our review) and highlight a pipeline for the development and evaluation of autonomous surgical robots. Key “building blocks” identified for development included surgical skill analysis, advanced surgical environment (ie, operative field) modeling, automatic control, and safe human–machine interface. Furthermore, Hashimoto and colleagues make an analogy with the recent development of autonomous cars (through integration of robotics, computer vision, and neural networks) as an example of how “synergistic reactions between different technologies can lead to unanticipated revolutionary technology”—much like the anticipated development of the next generation of AI-augmented surgical robotics.

At present, there are no specific dedicated frameworks or guidelines for development and evaluation in this emerging field. However, there are numerous relevant recent developments to note. For example, the IDEAL-D framework outlines the stages of device evaluation and regulation based on potential risk to patients.^11^ Ultimately, AI-augmented robotic systems, depending on their exact use, would likely fall into medium-risk or high-risk groups, and thus the device evaluation must be proportionate and rigorous—incorporating system, clinician, and patient assessments. Consensus-driven core outcome sets (COS) and core measurement instruments will be useful in standardizing these evaluation metrics. For example, the COHESIVE COS highlights outcomes for surgical procedures and devices across multiple domains but is more general and thus does not include AI-specific metrics.^27^ The RoboCOS Study is a similar COS initiative (in-process), specifically investigating outcomes in robot-assisted surgery, which may be helpful when completed (https://www.comet-initiative.org/Studies/Details/1608). TRIPOD-AI is a reporting guideline for the development and validation of predictive models based on AI, DECIDE-AI for the reporting of studies describing the early-stage clinical evaluation of decision support system based on AI, and SPIRIT/CONSORT-AI for randomized trials (and their protocols) evaluating intervention involving AI.^24,28–31^ However, all these initiatives are relatively general and do not cover many of the specificities of AI application for robotic surgery, which poses its own unique challenges.

### Strengths and Limitations

A scoping review design was chosen to capture the breadth of this evolving field, rather than explore items in-depth and exhaustively. As a result, the strengths of this study are its structured and wide search across literature databases and clinical trial registries. Screening was performed independently by at least 2 reviewers, as was data extraction. However, there are several limitations to this methodology. As with systematic reviews, there is a possibility that some relevant studies were missed, although it is unlikely these would have significantly altered the main messages of this study—which is, in essence, a call for rigorous and standardized evaluation, as well as structured reporting, in this field. This study is a review of the academic literature, and thus there is a possibility that there are AI applications for robotic surgery being developed or already developed for clinical settings in industry but not yet published (eg, for intellectual property reasons). Ideally, a more open-science approach will be adopted by further studies, which will accelerate the advancement of higher autonomy level systems. Finally, there are limitations to the primary data, which is currently too heterogenous and too small in quantity to perform any meaningful comparative meta-analysis. Numerous papers did not explicitly state key components or structures of the AI used.

## CONCLUSIONS

This scoping review provides an overview of the current landscape of intraoperative AI applications for robotic surgery in development, a rapidly expanding field. All included applications are in the preclinical (IDEAL stage 0) phase of development, with the majority operating with the lowest level of autonomy. The evaluation of these devices and the reporting of study findings are considerably heterogenous. Future work should focus on rigorous and standardized evaluation, structured reporting in this field, and the safe synergy of these technologies for higher autonomy applications.

## Supplementary Material
