# Supplementary material for: Intraoperative Applications of Artificial Intelligence in Robotic Surgery: A Scoping Review of Current Development Stages and Levels of Autonomy
Source: Ann Surg. 2022 Sep 30;278(6):896–903. doi: 10.1097/SLA.0000000000005700 (PMC10631501; doi:10.1097/SLA.0000000000005700)
Supplement: Supplementary file 1 [file sla-278-00896-s001.pdf]

SUPPLEMENTARY MATERIALS

Intraoperative applications of artificial intelligence in robotic surgery: a scoping review of current development stages and levels of autonomy

B. Vasey[1,2,3,*], K.A.N. Lippert[4,*], D.Z. Khan[5, 6], M.Ibrahim[2,8], C.H. Koh[5, 6], H. Layard Horsfall[5, 6], K.S. Lee[7], S. Williams[5,6], H.J. Marcus[5, 6, §], P. McCulloch[1, §].

[1.] Nuffield Department of Surgical Sciences, University of Oxford, Oxford, UK

[2.] Institute of Biomedical Engineering, Department of Engineering Science, University of Oxford, Oxford, UK

[3.] Critical Care Research Group, Nuffield Department of Clinical Neurosciences, University of Oxford, Oxford, UK

[4.] Institute of Psychiatry, Psychology and Neuroscience, King's College London, London, UK

[5.] Department of Neurosurgery, National Hospital for Neurology and Neurosurgery, London, UK.

[6.] Wellcome / EPSRC Centre for Interventional and Surgical Sciences, University College London, London, UK.

[7.] Bristol Medical School, Faculty of Health Sciences, University of Bristol, Bristol, UK.

[8.] Department of General Surgery, Maimonides Medical Center, Brooklyn, New York, USA.

[*] These authors contributed equally.

[§] These authors provided equal supervision.

Table of Contents

Supplementary Note 1: Search strategy for literature databases 2

Supplementary Note 2: Modified search strategy for trial registries 3

Supplementary Table 1 4

Supplementary Table 2 12

References 13

Supplementary Note 1: Search strategy for literature databases

1. (“artificial intelligence” or AI or “machine learning” or “deep learning” or “neural network” or “support vector machine” or “Bayesian network” or “nearest neighbour” or “random forest” or “pattern recognition” or “natural language processing” or (supervised adj2 learning) or (supervised adj2 learning) or “reinforced learning”).ab,kw,ti.
2. exp artificial intelligence/
3. exp Pattern Recognition, Automated/
4. (“surgical intervention” or “surgical procedure” or “surgical operation” or “surgical treatment” or “surgical technique” or “operative intervention” or “operative procedure” or “operative treatment” or “operative technique” or laparoscopic* or keyhole* or “minimally invasive surgery” or surgeon*).ab,kw,ti.
5. exp Surgical Procedures, Operative/
6. (robot* or mechatronic* or mechanic* or “Auris Surgical” or “CMR Surgical” or Versius or “Corindus Vascular” or CorPath or “Globus Medical” or AUTOBAHN or “Intuitive Surgical” or Da Vinci” or “Mazor Robotics” or Renaissance or Medrobotics or “Flex Robotic” or “Zimmer Biomet” or Rosa or Neocis or Yomi or “OMNI Ortho*” or OMNIBotics or Riverfield or Stereotaxis or Stryker or MAKO or “Rio Robotic” or MAKOPlasty or “THINK Surgical” or TSolution or “Titan Medical” or SPORT Surgical System” or TransEntrix or “Senhance Surgical” or “Virtual Incision” or AESOP or ZEUS or PUMA or Medtronic or “Verb Surgical” or Cori or Vicarious or Monteris or NeuroBlate or Brainlab or Accuray or “AVRA surgical” or Medrobotics or “Flex Robotix*NovaTract surgical” or Olympus).ab,kw,ti.
7. exp robotics/
8. exp disruptive technology/
9. exp Robotic Surgical Procedures/
10. (“telemedical surgery” or telesurgery or “remote surgery”).ab,kw,ti.
11. 1 or 2 or 3
12. 4 or 5
13. 6 or 7 or 8
14. 9 or 10
15. 12 and 13
16. 14 or 15
17. 11 and 16
18. limit 17 to yr=”2010-Current”
19. limit 18 to (editorial or letter or “review” or “systematic review”)
20. 18 not 19
21. limit 20 to conference abstract status [Limit not valid in Ovid MEDLINE(R),Ovid MEDLINE(R) Daily Update,Ovid MEDLINE(R) In-Process,Ovid MEDLINE(R) Publisher; records were retained]
22. limit 20 to yr=”2018-Current”
23. 20 not 21
24. limit 22 to conference abstracts [Limit not valid in Ovid MEDLINE(R),Ovid MEDLINE(R) Daily Update,Ovid MEDLINE(R) In-Process,Ovid MEDLINE(R) Publisher; records were retained]

Supplementary Note 2: Modified search strategy for trial registries

Cochrane

(artificial intelligence OR AI OR machine learning OR deep learning OR neural network OR support vector machine OR pattern recognition or natural language processing or reinforced learning or supervised learning) AND (*surg* OR surgical OR procedur* OR operat* OR intervention*) AND (*robot* OR robotic OR robotic surgery OR *mech* OR mechatronic)

ClinicalTrials.gov

(artificial intelligence OR machine learning OR deep learning OR neural network OR support vector OR recognition) AND (surgery OR surgical OR procedure OR operative OR intervention) AND (robot OR robotic OR robotic surgery OR mechanic OR mechatronic)

EU trials

(artificial intelligence OR machine learning OR deep learning OR neural network OR support vector OR recognition) AND (surgery OR surgical OR procedure OR operative OR intervention) AND (robot OR robotic OR robotic surgery OR mechanic OR mechatronic)

Supplementary Table 1

Publication	Speciality	Robotic platform	AI component's primary task	AI use category	AI model type	Training data type	IDEAL stage	Study type	Level* of autonomy	Outcome category
Abeywardena et al., 2019	Cross specialty	Da Vinci	Estimate tool-tissue force interactions	Robot control	NN	Force sensor	Stage 0	Preclinical - inorganic	Level 1	Regression
Ahmad et al., 2020 [1]	Obs-Gyne	Custom robot	Placental pose estimation	Enviroment modelling	NN	Videos	Stage 0	Preclinical - inorganic	Level 1	Object detection
Al-Abdullah et al., 2019 [2]	Cross speciality	NA	Estimating bone milling forces	Enviroment modelling	NN	Other (hardware)	Stage 0	Preclinical - inorganic	Level 1	Regression
Alambeigi et al., 2018 [3]	Cross speciality	Da Vinci	Automated tissue manipulation	Robot control	other	Kinematic & Images	Stage 0	Preclinical - organic tissue	Level 2	Efficacy
Amir-Khalili et al., 2014 [4]	Urology	NA	Identifying occluded vessels	Enviroment modelling	other	Videos	Stage 0	Preclinical - inorganic	Level 1	Classification
Antico et al., 2020 [5]	Trauma and Ortopedic	NA	Segmentation of femoral cartilage during knee arthorscopy	Enviroment modelling	NN	Images	Stage 0	Preclinical - inorganic	Level 1	Object detection
Antico et al., 2020 [6]	Trauma and Ortopedic	NA	Detect femoral cartilage during knee arthorscopy	Enviroment modelling	NN	Images	Stage 0	Preclinical - inorganic	Level 1	Object detection
Aviles et al., 2015 [7]	Cardiothoracic	Staubil RX60B Robot	Visual based force feedback estimation	Enviroment modelling	NN	Videos	Stage 0	Preclinical - inorganic	Level 1	Regression
Aviles et al., 2017 [8]	Cardiothoracic	Staubil RX60B Robot	Visual based force feedback estimation	Enviroment modelling	NN	Videos	Stage 0	Preclinical - inorganic	Level 1	Regression
Aviles-Rivero et al., 2018 [9]	Cardiothoracic	NA	Cardiac motion estimation	Enviroment modelling	other	Videos	Stage 0	Preclinical - inorganic	Level 1	Regression
Baek et al., 2018 [10]	General	APOLLON	Path planning around gall bladder to avoid collision and surrounding tissue	Planning/ navigation	RL	Images	Stage 0	Preclinical - inorganic	Level 1	Other
Baghdadi et al., 2019 [11]	Urology	NA	Evaluation of surgical performance	Skill analysis	LR	Videos	Stage 0	Preclinical - inorganic	Level 1	Classification
Baghdadi et al., 2020 [12]	Cross speciality	neuroArmPLUS	Surgeon skill prediction	Skill analysis	RF + NNC + LR	Videos	Stage 0	Preclinical - inorganic	Level 1	Classification
Bareum et al., 2017 [13]	General	NA	Detect surgical tools	Event detection	NN	Videos	Stage 0	Preclinical - inorganic	Level 1	Object detection
Berthet-Rayne et al., 2016 [14]	Obs-Gyne	Raven II	Assisting motions and evaluating performance	Robot control	HMM	Videos	Stage 0	Preclinical - inorganic	Level 1	Efficacy

Britz et al., 2019 [15]	Neurosurgery	CorPath GRX	Catheter position control	Robot control	unclear	NA	Stage 0	Preclinical - organic tissue	Level 1	Efficacy
Cai et al., 2020 [16]	Cross speciality	NA	Surgical instruments detection	Event detection	NN	Videos	Stage 0	Preclinical - inorganic	Level 1	Object detection
Calinon et al., 2014 [17]	General	STIFF-FLOP robot	Assisting motions	Robot control	GMM	Kinematic	Stage 0	Preclinical - inorganic	Level 2	Other
Cavallo et al., 2014 [18]	Cross speciality	LapSim Basic Skills 3.0	Evaluation of surgical performance	Skill analysis	HMM	Kinematic	Stage 0	Preclinical - inorganic	Level 1	Classification
Chen et al., 2020 [19]	Cross speciality	NA	Digital surgical smoke removal	Event detection	NN	Videos	Stage 0	Preclinical - inorganic	Level 1	Regression
Chi et al., 2018 [20]	Vascular	Custom robot	Catheterization motion modelling and trajectory planning	Planning/ navigation	GMM	Kinematic	Stage 0	Preclinical - inorganic	Level 2	Efficacy
Cho et al., 2021	Cross specialty	NA	Automated tip detection of surgical instruments	Tracking/ localisation	NN	Images	Stage 0	Preclinical - inorganic	Level 1	Object detection
De Momi et al., 2010 [21]	Neurosurgery	ROBOCAST/ MARS	Path planning	Planning/ navigation	other	Images	Stage 0	Preclinical - inorganic	Level 3	Efficacy
DiPietro et al., 2019 [22]	Cross speciality	Da Vinci	Segementing and classifying surgical tasks	Event detection	NN	Kinematic	Stage 0	Preclinical - inorganic	Level 1	Classification
Du et al., 2017 [23]	Cross speciality	NA	Assisting motions	Robot control	other	Kinematic	Stage 0	Preclinical - inorganic	Level 1	Efficacy
Du et al., 2018 [24]	Cross speciality	Da Vinci	Instrument pose estimation	Tracking/ localisation	NN	Videos	Stage 0	Preclinical - inorganic	Level 1	Object detection
Ershad et al., 2019 [25]	Cross speciality	Da Vinci	User performance identification	Skill analysis	K-SVD + SVM	Kinematic	Stage 0	Preclinical - inorganic	Level 1	Classification
Fard et al., 2017[26]	Cross speciality	Da Vinci	Surgical skill classification	Skill analysis	NNC+ SVM	Kinematic	Stage 0	Preclinical - inorganic	Level 1	Classification
Fichera et al., 2013 [27]	Cross speciality	NA	Predicting the consequences of laser radiation on soft-tissue	Enviroment modelling	other	Other (simulation)	Stage 0	Preclinical - inorganic	Level 1	Regression
Funke et al., 2019 [28]	Cross speciality	NA	Surgical skills classification	Skill analysis	NN	Kinematic & Videos	Stage 0	Preclinical - inorganic	Level 1	Classification
Gao et al., 2021	Cross specialty	Da Vinci	Detection and compensation for hysteretic forces in robotic joints	Robot control	NN	Kinematics	Stage 0	Preclinical - inorganic	Level 1	Regression
Gessert et al., 2018 [29]	Cross speciality	Hexapod robot	Pose estimation and tracking	Tracking/ localisation	NN	Images	Stage 0	Preclinical - inorganic	Level 1	Object detection
Gessert et al., 2020 [30]	Cross speciality	Hexapod robot	Force estimation	Enviroment modelling	NN	Images	Stage 0	Preclinical - inorganic	Level 1	Regression
Giataganas et al., 2013[31]	Cross speciality	KUKA	Improving accuray and stability of probe-tissue contact	Robot control	GMM	Images	Stage 0	Preclinical - inorganic	Level 1	Regression

Hattab et al., 2019 [32]	Urology	NA	Kidney edge detection	Enviroment modelling	NN	Images	Stage 0	Preclinical - inorganic	Level 1	Object detection
He et al., 2018 [33]	Ophtalmology	SHER	Classifying scleral forces	Enviroment modelling	NN	Images	Stage 0	Preclinical - inorganic	Level 1	Classification
He et al., 2019 [34]	Ophtalmology	SHER	Predicting force safety	Enviroment modelling	NN	Force sensor	Stage 0	Preclinical - inorganic	Level 1	Classification
He et al., 2020 [35]	Ophtalmology	SHER	Predicting scleral force	Enviroment modelling	NN	Force sensor	Stage 0	Preclinical - inorganic	Level 1	Classification
He et al., 2020	Ophthalmology	SHER	Predicting scleral force	Environment modelling	NN	Force sensor	Stage 0	Preclinical - inorganic	Level 1	Classification
Hong et al., 2019 [36]	Cross speciality	Da Vinci	Head motion classification	Tracking/ localisation	SVM	Force sensor	Stage 0	Preclinical - inorganic	Level 1	Classification
Itzkovich et al., 2022	Cross specialty	Da Vinci	Gesture classification in robotic suturing	Event detection	NN	Kinematic & Videos	Stage 0	Preclinical - inorganic	Level 1	Classification
Iyengar et al., 2020 [37]	Obs-Gyne	NA	Tube robot control	Enviroment modelling	RL	NA	Stage 0	Preclinical - inorganic	Level 1	Regression
Jing et al., 2020 [38]	Cross speciality	Not stated	Estimation of kinematic performance	Skill analysis	other	Kinematic	Stage 0	Preclinical - inorganic	Level 1	Efficacy
Jog et al., 2013 [39]	Cross speciality	Da Vinci	User trajectory evaluation	Planning/ navigation	SVM	Other (simulation)	Stage 0	Preclinical - inorganic	Level 1	Classification
Karmul Hassan et al., 2019 [40]	Cross speciality	NA	Surgical tools segementation	Event detection	NN + NNC	Videos	Stage 0	Preclinical - inorganic	Level 1	Object detection
Kassahun et al., 2013 [41]	Cardiothoracic	NA	Predicting catheter-aorta interaction	Enviroment modelling	GMM	Images	Stage 0	Preclinical - inorganic	Level 1	Regression
Khalid et al., 2020 [42]	Cross speciality	Da Vinci	Surgical actions detection	Event detection	NN	Videos	Stage 0	Preclinical - inorganic	Level 1	Classification
Kumazu et al., 2021	Cross specialty	Da Vinci	Automated segmentation of connective tissue fibres to define dissection planes	Environment modelling	NN	Images	Stage 0	Preclinical - inorganic	Level 1	Regression
Kuntz et al., 2020 [43]	Cross speciality	NA	Estimating shape of the tube robot	Tracking/ localisation	NN	Images	Stage 0	Preclinical - inorganic	Level 1	Regression
Lee et al., 2020 [44]	Otolaryngology	Da Vinci	Surgical instruments tracking	Tracking/ localisation	NN	Images	Stage 0	Preclinical - inorganic	Level 1	Object detection
Lee et al., 2020 [45]	Cross speciality	NA	Segmentation	Enviroment modelling	NN	Videos	Stage 0	Preclinical - inorganic	Level 1	Regression
Lei et al., 2021	General Surgery	UR5 robotic arm + custom puncture mechanism	Automated registration and tumour localisation for robot-assisted CT-guided puncture	Environment modelling	SVM	Kinematics	Stage 0	Preclinical - inorganic	Level 2	Regression

Li et al., 2021	Neurosurgery	NA	Trajectory and grinding speed planning for robot-assisted laminectomy	Planning/ navigation	NN + GMM	Images	Stage 0	Preclinical - organic tissue	Level 1	Efficacy
Li et al., 2021	Neurosurgery	NA	Laminar segmentation to enable trajectory planning	Environment modelling	NN	Images	Stage 0	Preclinical - inorganic	Level 1	Object detection
Liang et al., 2020 [46]	Cross speciality	NA	Position compensation and kinetic analysis	Robot control	SVM	Images	Stage 0	Preclinical - inorganic	Level 1	Regression
Lópes-Casado et al., 2019 [47]	Cross speciality	WAM manipulator and UR3 manipulator	Hand gesture recognition	Event detection	RL	NA	Stage 0	Preclinical - inorganic	Level 1	Classification
Luo et al., 2019 [48]	Urology	Da Vinci	Organ depth estimation	Enviroment modelling	NN	Images	Stage 0	Preclinical - inorganic	Level 1	Regression
Luongo et al., 2020 [49]	Urology	NA	Gesture identification	Event detection	NN	Videos	Stage 0	Preclinical - inorganic	Level 1	Classification
Marban et al., 2018 [50]	Cross speciality	NA	Force feeback	Enviroment modelling	NN	Videos	Stage 0	Preclinical - inorganic	Level 1	Regression
Marsden et al., 2020 [51]	Otolaryngology	Da Vinci	Cancer margin assessment	Enviroment modelling	NN	Images	Stage 0	Preclinical - inorganic	Level 1	Classification
Mikada et al., 2019 [52]	Cross speciality	PHANTOM (Master)	Estimating posture of surgical instuments	Tracking/ localisation	NN	Images	Stage 0	Preclinical - inorganic	Level 1	Object detection
Moccia et al., 2018 [53]	Neurosurgery	Micron handheld surgical robot	Vascular segmentation	Enviroment modelling	NN	Images	Stage 0	Preclinical - inorganic	Level 1	Object detection
Mohamadipanah et al., 2015 [54]	Cardiothoraric	NA	Cardiac motion compensation	Robot control	NN	Videos	Stage 0	Preclinical - inorganic	Level 1	Regression
Murali et al., 2015 [55]	Cross speciality	Da Vinci	Debridement and cutting of tissue	Robot control	unclear	Kinematic & Videos	Stage 0	Preclinical - inorganic	Level 2	Efficacy
Nakawala et al., 2018 [56]	Urology	NA	Current and next surgical steps recognition	Event detection	NN	Videos	Stage 0	Preclinical - inorganic	Level 1	Classification
Narayan et al., 2018 [57]	Cross speciality	NA	Trajectory planning	Planning/ navigation	NN	NA	Stage 0	Preclinical - inorganic	Level 2	Efficacy
Nazir et al., 2020 [58]	General	NA	Localization of intraoperative liver anatomy	Enviroment modelling	NN	Images	Stage 0	Preclinical - inorganic	Level 1	Efficacy
Nguyen et al., 2019 [59]	Cross speciality	NA	Surgical skill levels classification	Skill analysis	NN	Kinematic	Stage 0	Preclinical - inorganic	Level 1	Classification
Nguyen et al., 2019 [60]	Cross speciality	NA	Surgical cutting	Robot control	RL (NN)	NA	Stage 0	Preclinical - inorganic	Level 2	Efficacy
Nichols et al., 2013 [61]	Cross speciality	NA	Tissues identification and classification	Enviroment modelling	LR + GDA + SVM	Images	Stage 0	Preclinical - inorganic	Level 1	Classification

Nosrati et al., 2014 [62]	Urology	Da Vinci	Tissue segmentation	Enviroment modelling	RF	Videos	Stage 0	Preclinical - inorganic	Level 2	Object detection
Nosrati et al., 2016 [63]	Urology	Da Vinci	Endoscopic scene labelling and augmentation	Enviroment modelling	RF	Videos	Stage 0	Preclinical - inorganic	Level 2	Object detection
Omisore et al., 2019 [64]	Cardiothoraric	NA	Estimation of hysteresis	Enviroment modelling	NN	Kinematic & Force sensor	Stage 0	Preclinical - inorganic	Level 1	Regression
Omisore et al., 2021	Cross specialty	Custom robot	Motion control of flexible robots	Robot control	NN	Kinematics	Stage 0	Preclinical - inorganic	Level 1	Regression
Osa et al., 2013 [65]	Cross speciality	NA	Force feeback and risk of perforation detection	Enviroment modelling	SVM	Kinematic & Force sensor	Stage 0	Preclinical - inorganic	Level 1	Regression
Osa et al., 2014 [66]	Cross speciality	NA	Learning time and space-dependent trajectories	Planning/ navigation	other	Kinematic	Stage 0	Preclinical - inorganic	Level 2	Efficacy
Ostler et al., 2020 [67]	Cross speciality	NA	Acoustic signal analyis	Enviroment modelling	NN	Audio	Stage 0	Preclinical - organic tissue	Level 1	Classification
Padovan et al., 2021	Urology	NA	Real-time determination of the position and rotation of a target organ	Environment modelling	NN	Videos	Stage 0	Preclinical - inorganic	Level 1	Object detection
Padoy et al., 2011 [68]	Cross speciality	Da Vinci	Recognition of task completion and execution of next motion	Event detection	HMM	Kinematic	Stage 0	Preclinical - inorganic	Level 2	Classification
Padoy et al., 2011 [69]	Cross speciality	Da Vinci	Trajectories analysis	Planning/ navigation	unclear	Kinematic	Stage 0	Preclinical - inorganic	Level 2	Efficacy
Pahlavan et al., 2013 [70]	Vascular	Custom robot	Tactile sensing and feedback	Enviroment modelling	other	Kinematic & Force sensor	Stage 0	Preclinical - inorganic	Level 2	Regression
Power et al., 2015 [71]	Cross speciality	Raven II	Real-time learned task recognition and generating setpoint trajectories for haptic guidance	Planning/ navigation	HMM	Kinematic	Stage 0	Preclinical - inorganic	Level 2	Efficacy
Qiu et al., 2019 [72]	Cross speciality	Da Vinci	Tracking surgical tools location	Tracking/ localisation	NN + GMM	Videos	Stage 0	Preclinical - inorganic	Level 1	Object detection
Rafii-Tari et al., 2013 [73]	Vascular	Dynamixel MX-28, RobotIS	Endovascular navigation	Planning/ navigation	GMM	Kinematic	Stage 0	Preclinical - inorganic	Level 2	Classification
Rafii-Tari et al., 2014 [74]	Vascular	NA	Prediction of future movements	Planning/ navigation	HMM	Kinematic	Stage 0	Preclinical - inorganic	Level 2	Classification
Rafii-Tari et al., 2017 [75]	Vascular	NA	Endovascular navigation	Planning/ navigation	SVM	Kinematic & Force sensor	Stage 0	Preclinical - inorganic	Level 2	Classification
Ravasio et al., 2020 [76]	Ophtalmology	NA	Intra-operative tracking of the retinal fundus	Tracking/ localisation	NN	Images and Videos	Stage 0	Preclinical - inorganic	Level 1	Regression
Reiley et al., 2010 [77]	Cross speciality	Da Vinci	Defining trajectories for suturing, knot tying, and needle passing	Planning/ navigation	GMM	Kinematic	Stage 0	Preclinical - inorganic	Level 2	Classification

Reiter et al., 2012 [78]	Cross speciality	Da Vinci	Classifying and tracking surgical tools	Tracking/ localisation	GMM	Videos	Stage 0	Preclinical - inorganic	Level 1	Object detection
Rivas-Blanco et al., 2017 [79]	General	CISOBOT	Camera navigation	Robot control	RL (NN)	NA	Stage 0	Preclinical - in vivo	Level 2	Efficacy
Sachdeva et al., 2020 [80]	Cross speciality	NA	Robotic limbs rocognition	Tracking/ localisation	NN	Videos	Stage 0	Preclinical - inorganic	Level 1	Object detection
Saeidi et al., 2022	General Surgery	Smart Tissue Autonomous Robot (STAR)	Performing intestinal anastomosis	Robot control	NN	Kinematic & Images	Stage 0	Preclinical - in vivo	Level 2	Efficacy
Sang et al., 2016 [81]	Cross speciality	PHANTOM (Master)	Overcoming stick–slip behavior and suppressing vibration of surgical instruments	Robot control	NN	Kinematic	Stage 0	Preclinical - inorganic	Level 1	Efficacy
Sani et al., 2021	Cross specialty	NA	Estimating tool actions based on real-time data from hand actions	Robot control	NN	Kinematics	Stage 0	Preclinical - inorganic	Level 1	Efficacy
Sarikaya et al., 2017 [82]	Cross speciality	NA	Tools detection and localization	Tracking/ localisation	NN	Videos	Stage 0	Preclinical - inorganic	Level 1	Object detection
Sarikaya et al., 2018 [83]	Cross speciality	NA	Classification of gestures and surgical tasks	Event detection	NN	Kinematic & Videos	Stage 0	Preclinical - inorganic	Level 1	Classification
Schulman et al., 2013 [84]	Cross speciality	Raven II	Reproducing trajectories	Planning/ navigation	GMM	Kinematic	Stage 0	Preclinical - inorganic	Level 2	Regression
Shademan et al., 2016 [85]	General	Smart Tissue Autonomous Robot (STAR)	Anastomosis suturing	Robot control	unclear	NA	Stage 0	Preclinical - in vivo	Level 2	Efficacy
Shen et al., 2012 [86]	Cardiothoracic	NA	Tissues differentiation	Enviroment modelling	other	Force sensor	Stage 0	Preclinical - organic tissue	Level 1	Classification
Shen et al., 2019 [87]	Vascular	Omega 3	Guidewire tracking	Tracking/ localisation	NN	Kinematic	Stage 0	Preclinical - in vivo	Level 1	Efficacy
Stephens et al., 2019 [88]	Oral and Maxillofacial	Da Vinci	Grip force and jaw angle estimation	Tracking/ localisation	NN	Kinematic	Stage 0	Preclinical - inorganic	Level 1	Regression
Stroop et al., 2018 [89]	Cross speciality	Custom robot	Tissue differentiation	Enviroment modelling	other	Force sensor	Stage 0	Preclinical - organic tissue	Level 1	Clustering
Su et al., 2019 [90]	Cross speciality	LWR4+, KUKA	Shape cutting	Robot control	NN	Kinematic	Stage 0	Preclinical - inorganic	Level 1	Efficacy
Su et al., 2019 [91]	Cross speciality	LWR4+, KUKA	Tool detection, identification and feedback	Event detection	NN	Kinematic	Stage 0	Preclinical - inorganic	Level 1	Object detection
Su et al., 2020 [92]	Cross speciality	LWR4+, KUKA	Reproducing trajectories	Planning/ navigation	NN	Kinematic	Stage 0	Preclinical - inorganic	Level 2	Efficacy
Su et al., 2021	Cross specialty	Custom robot	Aspirating blood from the operative field	Event detection	NN	Images	Stage 0	Preclinical - organic tissue	Level 2	Other

Sun et al., 2018 [93]	Cross speciality	Denso Robotics	Medical instrument tracking	Tracking/ localisation	NN	Kinematic	Stage 0	Preclinical - organic tissue	Level 1	Object detection
Sun et al., 2019 [94]	General	HQ-2	Laparoscope and field of view control	Tracking/ localisation	other	Kinematic	Stage 0	Preclinical - inorganic	Level 2	Object detection
Sznitman et al., 2012 [95]	Cross speciality	NA	Instruments tracking	Tracking/ localisation	unclear	Videos	Stage 0	Preclinical - inorganic	Level 1	Object detection
Tan et al., 2019 [96]	Cross speciality	NA	Path planning	Planning/ navigation	RL (NN)	NA	Stage 0	Preclinical - inorganic	Level 3	Efficacy
Tanzi et al., 2021	Urology	NA	Segmentation of catheter and overlaying of 3D augmented reality image	Environment modelling	NN	Images	Stage 0	Preclinical - inorganic	Level 1	Object detection
Tao et al., 2012 [97]	Cross speciality	Da Vinci	Surgical gestures and skill levels classification	Skill analysis	K-SVD + HMM	Kinematic & Videos	Stage 0	Preclinical - inorganic	Level 1	Classification
Tao et al., 2013 [98]	Cross speciality	Da Vinci	Surgical gestures segmentation and recognition	Event detection	other	Kinematic & Videos	Stage 0	Preclinical - inorganic	Level 1	Classification
Tatinati et al., 2015 [99]	Cross speciality	NA	Tremor prediction and compensation	Robot control	SVM	Kinematic	Stage 0	Preclinical - inorganic	Level 1	Regression
Thananjeyan et al., 2017 [100]	Cross speciality	Da Vinci	Optimal tensioning and pattern cutting	Robot control	RL (NN)	NA	Stage 0	Preclinical - inorganic	Level 2	Efficacy
Torun et al., 2020 [101]	Trauma and Ortopedic	KUKA	Breakthrough detection	Event detection	NNC	Other (hardware)	Stage 0	Preclinical - organic tissue	Level 1	Classification
van den Berg et al., 2010 [102]	Cross speciality	Berkeley Surgical Robots	Defining and executing trajectories	Planning/ navigation	EM	Kinematic	Stage 0	Preclinical - inorganic	Level 2	Efficacy
Wang et al., 2018 [103]	Cross speciality	Da Vinci	Surgical skill level and surgical task recognition	Skill analysis	NN	Kinematic & Videos	Stage 0	Preclinical - inorganic	Level 1	Classification
Wang et al., 2018 [104]	Cross speciality	Da Vinci	Surgical skill level assessment	Skill analysis	NN	Kinematic & Videos	Stage 0	Preclinical - inorganic	Level 1	Classification
Weede et al., 2011 [105]	Cross speciality	KUKA + Stäubli RX90	Automated endoscopic camera positioning	Tracking/ localisation	HC + MLC + MM	Kinematic	Stage 0	Preclinical - inorganic	Level 2	Efficacy
Wesierski et al., 2018 [106]	Cross speciality	Da Vinci and others	Surgical instrument articulated pose estimation	Tracking/ localisation	SVM	Videos	Stage 0	Preclinical - inorganic	Level 1	Object detection
Wu et al., 2020 [107]	Cross speciality	Da Vinci	Perceived workload classification	Enviroment modelling	NBC	Videos & Other	Stage 0	Preclinical - inorganic	Level 1	Classification
Wu et al., 2021	Cross specialty	Da Vinci	Prediction of surgical gestures	Event detection	NN	Kinematic & Videos	Stage 0	Preclinical - inorganic	Level 1	Classification
Xu et al., 2019 [108]	General	NA	Depth map estimation from 2D images	Enviroment modelling	NN	Videos	Stage 0	Preclinical - inorganic	Level 1	Regression
Yu et al., 2020 [109]	Cross speciality	Da Vinci	Surgical instrument detection	Event detection	NN	Videos	Stage 0	Preclinical - inorganic	Level 1	Object detection

Zhang et al., 2020 [110]	General	NA	Tracking of surgical instruments	Tracking/ localisation	NN	Videos	Stage 0	Preclinical - inorganic	Level 2	Object detection
Zhao et al., 2019 [111]	Vascular	NA	Endovascular navigation	Planning/ navigation	NN	Videos & Force sensor	Stage 0	Preclinical - inorganic	Level 2	Efficacy
Zhao et al., 2019[112]	General	NA	Surgical instruments detection	Tracking/ localisation	NN	Videos	Stage 0	Preclinical - inorganic	Level 1	Object detection
Zia et al., 2019 [113]	Urology	Da Vinci	Surgical steps recognition	Skill analysis	NN	Videos	Stage 0	Preclinical - inorganic	Level 1	Classification

Summary table of included studies. *defined after the Yang *et al*. classification, no application at level 4 and above were retrieved; AI = artificial intelligence; EM = Expectation maximisation; GDA = Gaussian Discriminant Analysis; GMM = Gaussian Mixture Model; HC = Hierarchical Clustering; HMM = Hidden Markov Model; LR = logistic regression; MDP = Markov Decision Process; MLC = Maximum Likelihood Classifier; MM = Markov Model; NA = non available; NBC = Naive Bayes Classifier; NN = Neural Network (including feed-forward network, recurrent neural network, convolutional neural network, long short-term memory network, generative adversarial network, fuzzy network); NNC = Nearest Neighbour Classifier; RF = Random Forest; RL = Reinforcement Learning (including different type of algorithms); RT = Random Tree; SVM = Support Vector Machine

Supplementary Table 2

AI application		Environment modelling			Event detection			Planning & navigation			Robot control			Skill analysis			Tracking & localisation		
Level of autonomy*		1	2	3	1	2	3	1	2	3	1	2	3	1	2	3	1	2	3
Outcome category	***Outcome subcategory***																		
AI	*Classification*	10	0	0	10	1	0	1	4	0	0	0	0	11	0	0	1	0	0
AI	*Object detection*	8	2	0	5	0	0	0	0	0	0	0	0	0	0	0	13	2	0
AI	*Regression*	15	2	0	1	0	0	0	1	0	7	0	0	0	0	0	3	0	0
AI	*Other*	0	0	0	0	1	0	1	0	0	0	1	0	0	0	0	0	0	0
AI	*Clustering*	1	0	0	0	0	0	0	0	0	0	0	0	0	0	0	0	0	0
Procedural	*Efficacy*	1	0	0	0	0	0	1	8	2	6	7	0	1	0	0	1	1	0

Supplementary table 1: Outcome category by application of AI and level of autonomy. * defined after the Yang *et al*. classification, no application at level 4 and above were retrieved; AI = artificial intelligence.

References

1 Ahmad, M. A. *et al.* Deep learning-based monocular placental pose estimation: towards collaborative robotics in fetoscopy. *Int J Comput Assist Radiol Surg* 15, 1561-1571, doi:10.1007/s11548-020-02166-3 (2020).

2 Al-Abdullah, K. I., Lim, C. P., Najdovski, Z. & Yassin, W. A model-based bone milling state identification method via force sensing for a robotic surgical system. *Int J Med Robot* 15, e1989, doi:10.1002/rcs.1989 (2019).

3 Alambeigi, F., Wang, Z., Liu, Y. H., Taylor, R. H. & Armand, M. Toward Semi-autonomous Cryoablation of Kidney Tumors via Model-Independent Deformable Tissue Manipulation Technique. *Ann Biomed Eng* 46, 1650-1662, doi:10.1007/s10439-018-2074-y (2018).

4 Amir-Khalili, A. *et al.* Auto localization and segmentation of occluded vessels in robot-assisted partial nephrectomy. *Med Image Comput Comput Assist Interv* 17, 407-414, doi:10.1007/978-3-319-10404-1_51 (2014).

5 Antico, M. *et al.* Deep Learning for US Image Quality Assessment Based on Femoral Cartilage Boundary Detection in Autonomous Knee Arthroscopy. *IEEE Trans Ultrason Ferroelectr Freq Control* 67, 2543-2552, doi:10.1109/tuffc.2020.2965291 (2020).

6 Antico, M. *et al.* Deep Learning-Based Femoral Cartilage Automatic Segmentation in Ultrasound Imaging for Guidance in Robotic Knee Arthroscopy. *Ultrasound Med Biol* 46, 422-435, doi:10.1016/j.ultrasmedbio.2019.10.015 (2020).

7 Aviles, A. I., Alsaleh, S. M., Sobrevilla, P. & Casals, A. Force-feedback sensory substitution using supervised recurrent learning for robotic-assisted surgery. *Annu Int Conf IEEE Eng Med Biol Soc* 2015, 1-4, doi:10.1109/embc.2015.7318246 (2015).

8 Aviles, A. I., Alsaleh, S. M., Hahn, J. K. & Casals, A. Towards Retrieving Force Feedback in Robotic-Assisted Surgery: A Supervised Neuro-Recurrent-Vision Approach. *IEEE Transactions on Haptics* 10, 431-443, doi:10.1109/TOH.2016.2640289 (2017).

9 Aviles-Rivero, A. I., Alsaleh, S. M. & Casals, A. Sliding to predict: vision-based beating heart motion estimation by modeling temporal interactions. *Int J Comput Assist Radiol Surg* 13, 353-361, doi:10.1007/s11548-018-1702-1 (2018).

10 Baek, D., Hwang, M., Kim, H. & Kwon, D. in *2018 15th International Conference on Ubiquitous Robots (UR).* 342-347.

11 Baghdadi, A., Hussein, A. A., Ahmed, Y., Cavuoto, L. A. & Guru, K. A. A computer vision technique for automated assessment of surgical performance using surgeons' console-feed videos. *Int J Comput Assist Radiol Surg* 14, 697-707, doi:10.1007/s11548-018-1881-9 (2019).

12 Baghdadi, A. *et al.* Data analytics interrogates robotic surgical performance using a microsurgery-specific haptic device. *Expert Review of Medical Devices* 17, 721-730, doi:10.1080/17434440.2020.1782736 (2020).

13 Bareum, C., Kyungmin, J., Songe, C. & Jaesoon, C. Surgical-tools detection based on Convolutional Neural Network in laparoscopic robot-assisted surgery. *Annu Int Conf IEEE Eng Med Biol Soc* 2017, 1756-1759, doi:10.1109/embc.2017.8037183 (2017).

14 Berthet-Rayne, P., Power, M., King, H. & Yang, G. Z. in *2016 IEEE International Conference on Robotics and Automation (ICRA).* 715-722.

15 Britz, G. W. *et al.* Neuroendovascular-specific engineering modifications to the CorPath GRX Robotic System. *J Neurosurg*, 1-7, doi:10.3171/2019.9.Jns192113 (2019).

16 Cai, T. & Zhao, Z. Convolutional neural network-based surgical instrument detection. *Technol Health Care* 28, 81-88, doi:10.3233/thc-209009 (2020).

17 Calinon, S., Bruno, D., Malekzadeh, M. S., Nanayakkara, T. & Caldwell, D. G. Human–robot skills transfer interfaces for a flexible surgical robot. *Computer Methods and Programs in Biomedicine* 116, 81-96, doi:https://doi.org/10.1016/j.cmpb.2013.12.015 (2014).

18 Cavallo, F. *et al.* Biomechanics-machine learning system for surgical gesture analysis and development of technologies for minimal access surgery. *Surg Innov* 21, 504-512, doi:10.1177/1553350613510612 (2014).

19 Chen, S. *et al.* Voxel-based morphometry analysis and machine learning based classification in pediatric mesial temporal lobe epilepsy with hippocampal sclerosis. *Brain Imaging and Behavior* 14, 1945-1954, doi:10.1007/s11682-019-00138-z (2020).

20 Chi, W. *et al.* Learning-based endovascular navigation through the use of non-rigid registration for collaborative robotic catheterization. *Int J Comput Assist Radiol Surg* 13, 855-864, doi:10.1007/s11548-018-1743-5 (2018).

21 De Momi, E. & Ferrigno, G. Robotic and artificial intelligence for keyhole neurosurgery: The ROBOCAST project, a multi-modal autonomous path planner. *Proceedings of the Institution of Mechanical Engineers, Part H: Journal of Engineering in Medicine* 224, 715-727, doi:10.1243/09544119JEIM585 (2010).

22 DiPietro, R. *et al.* Segmenting and classifying activities in robot-assisted surgery with recurrent neural networks. *Int J Comput Assist Radiol Surg* 14, 2005-2020, doi:10.1007/s11548-019-01953-x (2019).

23 Du, Z., Wang, W., Yan, Z., Dong, W. & Wang, W. Variable Admittance Control Based on Fuzzy Reinforcement Learning for Minimally Invasive Surgery Manipulator. *Sensors* 17, doi:10.3390/s17040844 (2017).

24 Du, X. *et al.* Articulated Multi-Instrument 2-D Pose Estimation Using Fully Convolutional Networks. *IEEE Transactions on Medical Imaging* 37, 1276-1287, doi:10.1109/TMI.2017.2787672 (2018).

25 Ershad, M., Rege, R. & Fey, A. M. Automatic and near real-time stylistic behavior assessment in robotic surgery. *Int J Comput Assist Radiol Surg* 14, 635-643, doi:10.1007/s11548-019-01920-6 (2019).

26 Fard, M. J. *et al.* Automated robot-assisted surgical skill evaluation: Predictive analytics approach. *Int J Med Robot* 14, doi:10.1002/rcs.1850 (2017).

27 Fichera, L., Pardo, D. & Mattos, L. *Modeling Tissue Temperature Dynamics during Laser Exposure*. Vol. 7903 (2013).

28 Funke, I., Mees, S., Weitz, J. & Speidel, S. *Video-based surgical skill assessment using 3D convolutional neural networks*. (2019).

29 Gessert, N., Schlüter, M. & Schlaefer, A. A deep learning approach for pose estimation from volumetric OCT data. *Medical Image Analysis* 46, 162 179 (2018).

30 Gessert, N., Bengs, M., Schlüter, M. & Schlaefer, A. *Deep learning with 4D spatio-temporal data representations for OCT-based force estimation*. (2020).

31 Giataganas, P., Vitiello, V., Simaiaki, V., Lopez, E. & Guang-Zhong, Y. in *2013 IEEE International Conference on Robotics and Automation.* 5378-5383.

32 Hattab, G. *et al.* Kidney edge detection in laparoscopic image data for computer-assisted surgery : Kidney edge detection. *Int J Comput Assist Radiol Surg* 15, 379-387, doi:10.1007/s11548-019-02102-0 (2019).

33 He, C., Patel, N., Ebrahimi, A., Kobilarov, M. & Iordachita, I. Preliminary study of an RNN-based active interventional robotic system (AIRS) in retinal microsurgery. *Int J Comput Assist Radiol Surg* 14, 945-954, doi:10.1007/s11548-019-01947-9 (2018).

34 He, C., Patel, N., Iordachita, I. & Kobilarov, M. Enabling Technology for Safe Robot-Assisted Retinal Surgery: Early Warning for Unsafe Scleral Force. *IEEE Int Conf Robot Autom* 2019, 3889-3894, doi:10.1109/icra.2019.8794427 (2019).

35 He, C. *et al.* Toward Safe Retinal Microsurgery: Development and Evaluation of an RNN-Based Active Interventional Control Framework. *IEEE Trans Biomed Eng* 67, 966-977, doi:10.1109/tbme.2019.2926060 (2020).

36 Hong, N., Kim, M., Lee, C. & Kim, S. Head-mounted interface for intuitive vision control and continuous surgical operation in a surgical robot system. *Med Biol Eng Comput* 57, 601-614, doi:10.1007/s11517-018-1902-4 (2019).

37 Iyengar, K., Dwyer, G. & Stoyanov, D. Investigating exploration for deep reinforcement learning of concentric tube robot control. *International journal of computer assisted radiology and surgery* 15, 1157-1165, doi:10.1007/s11548-020-02194-z (2020).

38 Jing, Y., Lingyan, J., Xinge, S., Deming, Z. & Ming, H. Dimensional Optimization for Minimally Invasive Surgery Robot Based on Double Space and Kinematic Accuracy Reliability Index. *Journal of Engineering and Science in Medical Diagnostics and Therapy* 3, doi:10.1115/1.4046382 (2020).

39 Jog, A. *et al.* in *2011 IEEE International Conference on Robotics and Automation.* 5273-5278.

40 Kamrul Hasan, S. M. & Linte, C. A. in *2019 41st Annual International Conference of the IEEE Engineering in Medicine and Biology Society (EMBC).* 7205-7211.

41 Kassahun, Y., Yu, B. & Vander Poorten, E. *Learning Catheter-Aorta Interaction Model Using Joint Probability Densities*. (2013).

42 Khalid, S., Goldenberg, M., Grantcharov, T., Taati, B. & Rudzicz, F. Evaluation of Deep Learning Models for Identifying Surgical Actions and Measuring Performance. *JAMA Netw Open* 3, e201664, doi:10.1001/jamanetworkopen.2020.1664 (2020).

43 Kuntz, A., Sethi, A., Webster, R. J. & Alterovitz, R. Learning the Complete Shape of Concentric Tube Robots. *IEEE Transactions on Medical Robotics and Bionics* 2, 140-147, doi:10.1109/TMRB.2020.2974523 (2020).

44 Lee, D. *et al.* Evaluation of Surgical Skills during Robotic Surgery by Deep Learning-Based Multiple Surgical Instrument Tracking in Training and Actual Operations. *J Clin Med* 9, doi:10.3390/jcm9061964 (2020).

45 Lee, J. Y. *et al.* Ultrasound needle segmentation and trajectory prediction using excitation network. *International Journal of Computer Assisted Radiology and Surgery* 15, 437-443, doi:10.1007/s11548-019-02113-x (2020).

46 Liang, Y., Du, Z., Wang, W. & Sun, L. A Novel Position Compensation Scheme for Cable-Pulley Mechanisms Used in Laparoscopic Surgical Robots. *Sensors (Basel)* 17, doi:10.3390/s17102257 (2020).

47 López-Casado, C., Bauzano, E., Rivas-Blanco, I., Pérez-del-Pulgar, C. J. & Muñoz, V. F. A Gesture Recognition Algorithm for Hand-Assisted Laparoscopic Surgery. *Sensors* 19, doi:10.3390/s19235182 (2019).

48 Luo, H., Hu, Q. & Jia, F. Details preserved unsupervised depth estimation by fusing traditional stereo knowledge from laparoscopic images. *Healthc Technol Lett* 6, 154-158, doi:10.1049/htl.2019.0063 (2019).

49 Luongo, F., Hakim, R., Nguyen, J. H., Anandkumar, A. & Hung, A. J. Deep learning-based computer vision to recognize and classify suturing gestures in robot-assisted surgery. *Surgery* 169, 1240-1244, doi:10.1016/j.surg.2020.08.016 (2020).

50 Marban, A., Srinivasan, V., Samek, W., Fernández, J. & Casals, A. A recurrent convolutional neural network approach for sensorless force estimation in robotic surgery. *Biomedical Signal Processing and Control* 50, 134-150, doi:https://doi.org/10.1016/j.bspc.2019.01.011 (2018).

51 Marsden, M. *et al.* Intraoperative Margin Assessment in Oral and Oropharyngeal Cancer Using Label-Free Fluorescence Lifetime Imaging and Machine Learning. *IEEE Transactions on Biomedical Engineering* 68, 857-868, doi:10.1109/TBME.2020.3010480 (2020).

52 Mikada, T., Kanno, T., Kawase, T., Miyazaki, T. & Kawashima, K. Three-dimensional posture estimation of robot forceps using endoscope with convolutional neural network. *Int J Med Robot* 16, e2062, doi:10.1002/rcs.2062 (2019).

53 Moccia, S. *et al.* Toward Improving Safety in Neurosurgery with an Active Handheld Instrument. *Ann Biomed Eng* 46, 1450-1464, doi:10.1007/s10439-018-2091-x (2018).

54 Mohamadipanah, H., Hoberock, L. L. & Andalibi, M. Predictive Model Reference Adaptive Controller to Compensate Heart Motion in Minimally Invasive CABG Surgery. *Cardiovascular Engineering and Technology* 6, 329-339, doi:10.1007/s13239-015-0225-y (2015).

55 Murali, A. *et al.* in *2015 IEEE International Conference on Robotics and Automation (ICRA).* 1202-1209.

56 Nakawala, H. *et al.* "Deep-Onto" network for surgical workflow and context recognition. *Int J Comput Assist Radiol Surg* 14, 685-696, doi:10.1007/s11548-018-1882-8 (2018).

57 Narayan, J., Singla, E., Soni, S. & Singla, A. Adaptive neuro-fuzzy inference system–based path planning of 5-degrees-of-freedom spatial manipulator for medical applications. *Proceedings of the Institution of Mechanical Engineers, Part H: Journal of Engineering in Medicine* 232, 726-732, doi:10.1177/0954411918781418 (2018).

58 Nazir, A. *et al.* SPST-CNN: Spatial pyramid based searching and tagging of liver's intraoperative live views via CNN for minimal invasive surgery. *J Biomed Inform* 106, 103430, doi:10.1016/j.jbi.2020.103430 (2020).

59 Nguyen, X. A., Ljuhar, D., Pacilli, M., Nataraja, R. M. & Chauhan, S. Surgical skill levels: Classification and analysis using deep neural network model and motion signals. *Computer Methods and Programs in Biomedicine* 177, 1-8, doi:https://doi.org/10.1016/j.cmpb.2019.05.008 (2019).

60 Nguyen, T. T., Nguyen, N. D., Bello, F. & Nahavandi, S. A New Tensioning Method using Deep Reinforcement Learning for Surgical Pattern Cutting. *2019 IEEE International Conference on Industrial Technology (ICIT)*, 1339-1344 (2019).

61 Nichols, K. A. & Okamura, A. M. in *2013 IEEE International Conference on Robotics and Automation.* 4384-4389.

62 Nosrati, M. S. *et al.* Efficient multi-organ segmentation in multi-view endoscopic videos using pre-operative priors. *Med Image Comput Comput Assist Interv* 17, 324-331, doi:10.1007/978-3-319-10470-6_41 (2014).

63 Nosrati, M. S. *et al.* Endoscopic scene labelling and augmentation using intraoperative pulsatile motion and colour appearance cues with preoperative anatomical priors. *Int J Comput Assist Radiol Surg* 11, 1409-1418, doi:10.1007/s11548-015-1331-x (2016).

64 Omisore, O. M. *et al.* in *2019 41st Annual International Conference of the IEEE Engineering in Medicine and Biology Society (EMBC).* 5399-5402.

65 Osa, T., Haniu, T., Harada, K., Sugita, N. & Mitsuishi, M. in *2013 IEEE/RSJ International Conference on Intelligent Robots and Systems.* 2572-2577.

66 Osa, T., Sugita, N. & Mitsuishi, M. *Online Trajectory Planning in Dynamic Environments for Surgical Task Automation*. (2014).

67 Ostler, D. *et al.* Acoustic signal analysis of instrument-tissue interaction for minimally invasive interventions. *Int J Comput Assist Radiol Surg* 15, 771-779, doi:10.1007/s11548-020-02146-7 (2020).

68 Padoy, N. & Hager, G. D. in *2011 IEEE International Conference on Robotics and Automation.* 5285-5292.

69 Padoy, N. & Hager, G. D. Spatio-temporal registration of multiple trajectories. *Med Image Comput Comput Assist Interv* 14, 145-152, doi:10.1007/978-3-642-23623-5_19 (2011).

70 Pahlavan, P., Najarian, S., Afshari, E. & Moini, M. Artery cross-clamping during laparoscopic vascular surgeries; a computational tactile sensing approach. *Biomed Mater Eng* 23, 423-432, doi:10.3233/bme-130764 (2013).

71 Power, M., Rafii-Tari, H., Bergeles, C., Vitiello, V. & Yang, G.-Z. in *2015 IEEE International Conference on Robotics and Automation (ICRA).* 5330-5337.

72 Qiu, L., Li, C. & Ren, H. Real-time surgical instrument tracking in robot-assisted surgery using multi-domain convolutional neural network. *Healthc Technol Lett* 6, 159-164, doi:10.1049/htl.2019.0068 (2019).

73 Rafii-Tari, H., Liu, J., Lee, S. L., Bicknell, C. & Yang, G. Z. Learning-based modeling of endovascular navigation for collaborative robotic catheterization. *Med Image Comput Comput Assist Interv* 16, 369-377, doi:10.1007/978-3-642-40763-5_46 (2013).

74 Rafii-Tari, H., Liu, J., Payne, C. J., Bicknell, C. & Yang, G. Z. Hierarchical HMM based learning of navigation primitives for cooperative robotic endovascular catheterization. *Med Image Comput Comput Assist Interv* 17, 496-503, doi:10.1007/978-3-319-10404-1_62 (2014).

75 Rafii-Tari, H. *et al.* Objective Assessment of Endovascular Navigation Skills with Force Sensing. *Ann Biomed Eng* 45, 1315-1327, doi:10.1007/s10439-017-1791-y (2017).

76 Ravasio, C. S. *et al.* Learned optical flow for intra-operative tracking of the retinal fundus. *Int J Comput Assist Radiol Surg* 15, 827-836, doi:10.1007/s11548-020-02160-9 (2020).

77 Reiley, C. E., Plaku, E. & Hager, G. D. in *2010 Annual International Conference of the IEEE Engineering in Medicine and Biology.* 967-970.

78 Reiter, A., Allen, P. K. & Zhao, T. Feature classification for tracking articulated surgical tools. *Med Image Comput Comput Assist Interv* 15, 592-600, doi:10.1007/978-3-642-33418-4_73 (2012).

79 Rivas-Blanco, I. *et al.* Smart Cable-Driven Camera Robotic Assistant. *IEEE Transactions on Human-Machine Systems* 48, 183-196, doi:10.1109/THMS.2017.2767286 (2017).

80 Sachdeva, N., Klopukh, M., Clair, R. & Hahn, W. *Using Conditional Generative Adversarial Networks to Reduce the Effects of Latency in Robotic Telesurgery*. (2020).

81 Sang, H., Yang, C., Liu, F., Yun, J. & Jin, G. A fuzzy neural network sliding mode controller for vibration suppression in robotically assisted minimally invasive surgery. *Int J Med Robot* 12, 670-679, doi:10.1002/rcs.1784 (2016).

82 Sarikaya, D., Corso, J. J. & Guru, K. A. Detection and Localization of Robotic Tools in Robot-Assisted Surgery Videos Using Deep Neural Networks for Region Proposal and Detection. *IEEE Transactions on Medical Imaging* 36, 1542-1549, doi:10.1109/TMI.2017.2665671 (2017).

83 Sarikaya, D., Guru, K. & Corso, J. *Joint Surgical Gesture and Task Classification with Multi-Task and Multimodal Learning*. (2018).

84 Schulman, J., Gupta, A., Venkatesan, S., Tayson-Frederick, M. & Abbeel, P. in *2013 IEEE/RSJ International Conference on Intelligent Robots and Systems.* 4111-4117.

85 Shademan, A. *et al.* Supervised autonomous robotic soft tissue surgery. *Sci Transl Med* 8, 337ra364, doi:10.1126/scitranslmed.aad9398 (2016).

86 Shen, J. J., Kalantari, M., Kovecses, J., Angeles, J. & Dargahi, J. Viscoelastic Modeling of the Contact Interaction Between a Tactile Sensor and an Atrial Tissue. *IEEE Transactions on Biomedical Engineering* 59, 1727-1738, doi:10.1109/TBME.2012.2193127 (2012).

87 Shen, H. *et al.* A novel robotic system for vascular intervention: principles, performances, and applications. *Int J Comput Assist Radiol Surg* 14, 671-683, doi:10.1007/s11548-018-01906-w (2019).

88 Stephens, T. K. *et al.* Conditions for reliable grip force and jaw angle estimation of da Vinci surgical tools. *International Journal of Computer Assisted Radiology and Surgery* 14, 117-127, doi:10.1007/s11548-018-1866-8 (2019).

89 Stroop, R., Nakamura, M., Schoukens, J. & Oliva Uribe, D. Tactile sensor-based real-time clustering for tissue differentiation. *International Journal of Computer Assisted Radiology and Surgery* 14, 129-137, doi:10.1007/s11548-018-1869-5 (2018).

90 Su, H. *et al.* Experimental validation of manipulability optimization control of a 7-DoF serial manipulator for robot-assisted surgery. *Int J Med Robot* 17, 1-11, doi:10.1002/rcs.2193 (2019).

91 Su, H. *et al.* Towards Model-Free Tool Dynamic Identification and Calibration Using Multi-Layer Neural Network. *Sensors* 19, 3636 (2019).

92 Su, H. *et al.* Improved recurrent neural network-based manipulator control with remote center of motion constraints: Experimental results. *Neural Networks* 131, 291-299, doi:https://doi.org/10.1016/j.neunet.2020.07.033 (2020).

93 Sun, Z., Maréchal, L. & Foong, S. Passive magnetic-based localization for precise untethered medical instrument tracking. *Computer Methods and Programs in Biomedicine* 156, 151-161, doi:https://doi.org/10.1016/j.cmpb.2017.12.018 (2018).

94 Sun, Y., Pan, B., Zou, S. & Fu, Y. Adaptive Fusion-Based Autonomous Laparoscope Control for Semi-Autonomous Surgery. *J Med Syst* 44, 4, doi:10.1007/s10916-019-1460-9 (2019).

95 Sznitman, R. *et al.* Data-driven visual tracking in retinal microsurgery. *Med Image Comput Comput Assist Interv* 15, 568-575, doi:10.1007/978-3-642-33418-4_70 (2012).

96 Tan, X., Lee, Y., Chng, C.-B., Lim, K.-B. & Chui, C.-K. Robot-assisted flexible needle insertion using universal distributional deep reinforcement learning. *International Journal of Computer Assisted Radiology and Surgery* 15, 341-349, doi:10.1007/s11548-019-02098-7 (2019).

97 Tao, L., Elhamifar, E., Khudanpur, S., Hager, G. D. & Vidal, R. in *Information Processing in Computer-Assisted Interventions.* (eds Purang Abolmaesumi, Leo Joskowicz, Nassir Navab, & Pierre Jannin) 167-177 (Springer Berlin Heidelberg).

98 Tao, L., Zappella, L., Hager, G. D. & Vidal, R. in *Medical Image Computing and Computer-Assisted Intervention – MICCAI 2013.* (eds Kensaku Mori *et al.*) 339-346 (Springer Berlin Heidelberg).

99 Tatinati, S., Veluvolu, K. C. & Ang, W. T. Multistep Prediction of Physiological Tremor Based on Machine Learning for Robotics Assisted Microsurgery. *IEEE Transactions on Cybernetics* 45, 328-339, doi:10.1109/TCYB.2014.2381495 (2015).

100 Thananjeyan, B. *et al.* in *2017 IEEE International Conference on Robotics and Automation (ICRA).* 2371-2378.

101 Torun, Y. & Öztürk, A. A New Breakthrough Detection Method for Bone Drilling in Robotic Orthopedic Surgery with Closed-Loop Control Approach. *Annals of Biomedical Engineering* 48, 1218-1229, doi:10.1007/s10439-019-02444-5 (2020).

102 van den Berg, J. *et al.* in *2010 IEEE International Conference on Robotics and Automation.* 2074-2081.

103 Wang, Z. & Fey, A. M. SATR-DL: Improving Surgical Skill Assessment And Task Recognition In Robot-Assisted Surgery With Deep Neural Networks. *2018 40th Annual International Conference of the IEEE Engineering in Medicine and Biology Society (EMBC)*, 1793-1796 (2018).

104 Wang, Z. & Fey, A. M. Deep learning with convolutional neural network for objective skill evaluation in robot-assisted surgery. *International Journal of Computer Assisted Radiology and Surgery* 13, 1959-1970, doi:10.1007/s11548-018-1860-1 (2018).

105 Weede, O., Mönnich, H., Müller, B. & Wörn, H. in *2011 IEEE International Conference on Robotics and Automation.* 5762-5768.

106 Wesierski, D. & Jezierska, A. Instrument detection and pose estimation with rigid part mixtures model in video-assisted surgeries. *Medical Image Analysis* 46, 244-265, doi:https://doi.org/10.1016/j.media.2018.03.012 (2018).

107 Wu, C. *et al.* Eye-Tracking Metrics Predict Perceived Workload in Robotic Surgical Skills Training. *Human Factors* 62, 1365-1386, doi:10.1177/0018720819874544 (2020).

108 Xu, K., Chen, Z. & Jia, F. Unsupervised binocular depth prediction network for laparoscopic surgery. *Computer Assisted Surgery* 24, 30-35, doi:10.1080/24699322.2018.1557889 (2019).

109 Yu, L., Wang, P., Yan, Y., Xia, Y. & Cao, W. MASSD: Multi-scale attention single shot detector for surgical instruments. *Computers in Biology and Medicine* 123, 103867, doi:https://doi.org/10.1016/j.compbiomed.2020.103867 (2020).

110 Zhang, J. & Gao, X. Object extraction via deep learning-based marker-free tracking framework of surgical instruments for laparoscope-holder robots. *International Journal of Computer Assisted Radiology and Surgery* 15, 1335-1345, doi:10.1007/s11548-020-02214-y (2020).

111 Zhao, Y. *et al.* A CNN-based prototype method of unstructured surgical state perception and navigation for an endovascular surgery robot. *Med Biol Eng Comput* 57, 1875-1887, doi:10.1007/s11517-019-02002-0 (2019).

112 Zhao, Z., Cai, T., Chang, F. & Cheng, X. Real-time surgical instrument detection in robot-assisted surgery using a convolutional neural network cascade. *Healthc Technol Lett* 6, 275-279, doi:10.1049/htl.2019.0064 (2019).

113 Zia, A., Guo, L., Zhou, L., Essa, I. & Jarc, A. Novel evaluation of surgical activity recognition models using task-based efficiency metrics. *International Journal of Computer Assisted Radiology and Surgery* 14, doi:10.1007/s11548-019-02025-w (2019).
